# Chronic active EBV infection in refractory enteritis with longitudinal ulcers with a cobblestone appearance: an autopsied case report

**DOI:** 10.1186/s12876-020-01589-1

**Published:** 2021-01-06

**Authors:** Yosuke Aihara, Kei Moriya, Naotaka Shimozato, Shinsaku Nagamatsu, Shinya Kobayashi, Masakazu Uejima, Hideki Matsuo, Eiwa Ishida, Hideo Yagi, Toshiya Nakatani, Hitoshi Yoshiji, Eiryo Kikuchi

**Affiliations:** 1 Department of Gastroenterology and Hepatology, Nara Prefecture General Medical Center, Nara, Japan; 2 Department of Gastroenterology and Hepatology, Nara Medical University, 840 Shijo-cho, Kashihara, Nara 634-8522 Japan; 3 Department of Hematology, Nara Prefecture General Medical Center, Nara, Japan; 4 Department of Pathology, Nara Prefecture General Medical Center, Nara, Japan

**Keywords:** Crohn’s disease, Cobblestone appearance, Inflammatory bowel disease, Refractory enteritis, Chronic active EBV infection, Lymphoproliferative disorders, Malignant lymphoma, Biologics, Capsule endoscopy, Case report

## Abstract

**Background:**

Chronic active Epstein–Barr virus infection (CAEBV) is defined as Epstein–Barr virus (EBV)-positive T/NK cell-related neoplasia, and its major clinical symptom is systemic inflammation presenting as infectious mononucleocytosis, whereas enteritis and diarrhea are minor clinical symptoms. The complex mixture of tumorigenic processes of EBV-positive cells and physical symptoms of systemic inflammatory disease constitutes the varied phenotypes of CAEBV. Herein, we describe a case of CAEBV that was initially diagnosed as Crohn’s disease (CD) based on ileal ulcers and clinical symptoms of enteritis.

**Case presentation:**

A 19-year-old woman complained of abdominal pain and fever. Blood examination showed normal blood cell counts without atypical lymphocyte but detected modest inflammation, hypoalbuminemia, slight liver dysfunction, and evidence of past EBV infection. The esophagogastroduodenoscopic findings were normal. However, colonoscopy revealed a few small ulcers in the terminal ileum. The jejunum and ileum also exhibited various forms of ulcers, exhibiting a cobblestone appearance, on capsule endoscopy. Based on these clinical findings, she was strongly suspected with CD. In the course of treatment by steroid and biologics for refractory enteritis, skin ulcers appeared about 50 months after her initial hospital visit. Immunohistology of her skin biopsy revealed proliferation of EBV-encoded small RNA (EBER)-positive atypical lymphocytes. We retrospectively assessed her previous ileal ulcer biopsy before treatment and found many EBER-positive lymphocytes. Blood EBV DNA was also positive. Therefore, she was diagnosed with extranodal NK/T-cell lymphoma with CAEBV-related enteritis rather than CD. She was treated with cyclosporine and prednisolone combination therapy for CAEBV-related systemic inflammation and chemotherapy for malignant lymphoma. Unfortunately, her disease continued to progress, leading to multiple organ failure and death at the age of 23 years.

**Conclusion:**

Clinicians need to remember the possibility of CAEBV as a differential diagnosis of refractory enteritis. Enteritis with intestinal ulcer is a rare symptom of CAEBV, and it is impossible to acquire a definitive diagnosis by ulcer morphology only. In cases where the possibility of CAEBV remains, tissue EBVR expression should be checked by in situ hybridization and blood EBV DNA.

## Background

Enteritis is an inflammatory disease of the small intestine caused by some factors, such as bacterial or viral infections, ischemia, vasculitis, chemical or radiological tissue damage, and immune disorders including inflammatory bowel disease (IBD), with phenotype variation [1]. In fact, few cases of chronic active Epstein–Barr virus (CAEBV) reveal enteritis [2–4]. Previously, CAEBV was thought to be a rare child disease limitedly occurring in the East Asia. However, CAEBV has been recently found to occur worldwide with no relation to patient’s age [5]. Although CAEBV is a phenotype of EBV-lymphoproliferative disorders, its major clinical symptom is systemic inflammation presenting as infectious mononucleosis (IM) and enteritis as well as diarrhea are minor clinical symptoms. The complex mixture of tumorigenic processes associated with EBV-positive cells and the physical symptoms of inflammatory systemic disease constitute the varied phenotypes of CAEBV. The median period between the first visit and the definite diagnosis of CAEBV was 20 months. This extended duration was mainly due to the need to first exclude other diseases or cases where patients were diagnosed with unknown fever [6]. Although CAEBV generally presents with IM-like symptoms, we recently experienced a case of a young woman with CAEBV that appeared with refractory enteritis and was indistinguishable from Crohn’s disease (CD). This report of an autopsied CAEBV case will be of educational help, given the difficulty in acquiring a definite diagnosis.

## Case presentation

**Fig. 1:**
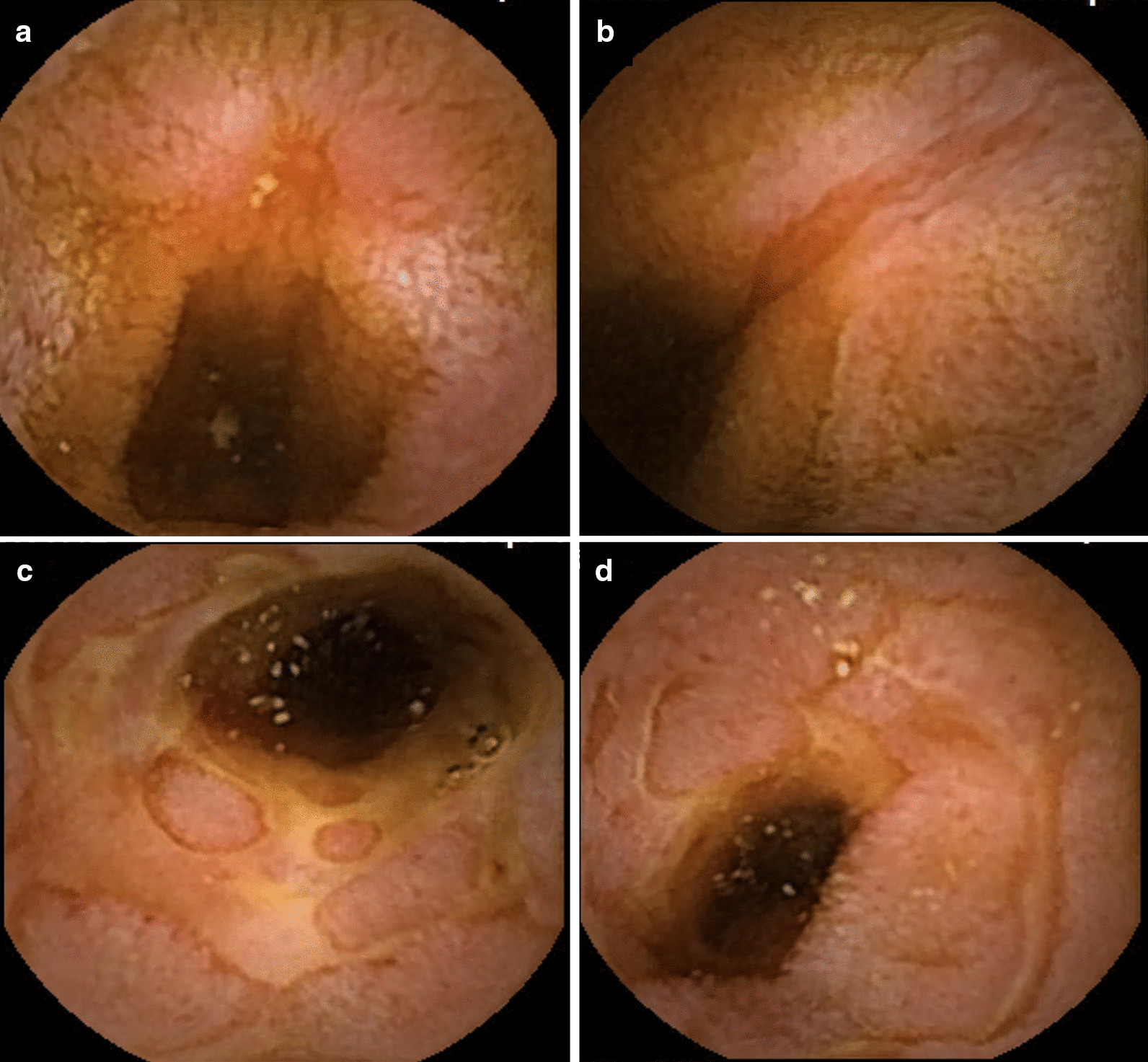
Various forms of ulcers on digestive organs detected by capsule endoscopy prior to treatment. The surrounding mucosa was free from inflammation. **a** Multiple aphthous ulcers on the jejunum. **b** Liner ulcers on the jejunum. **c** Longitudinal ulcers with a cobblestone appearance on the ileum. **d** Circular ulcers on ileum

**Fig. 2:**
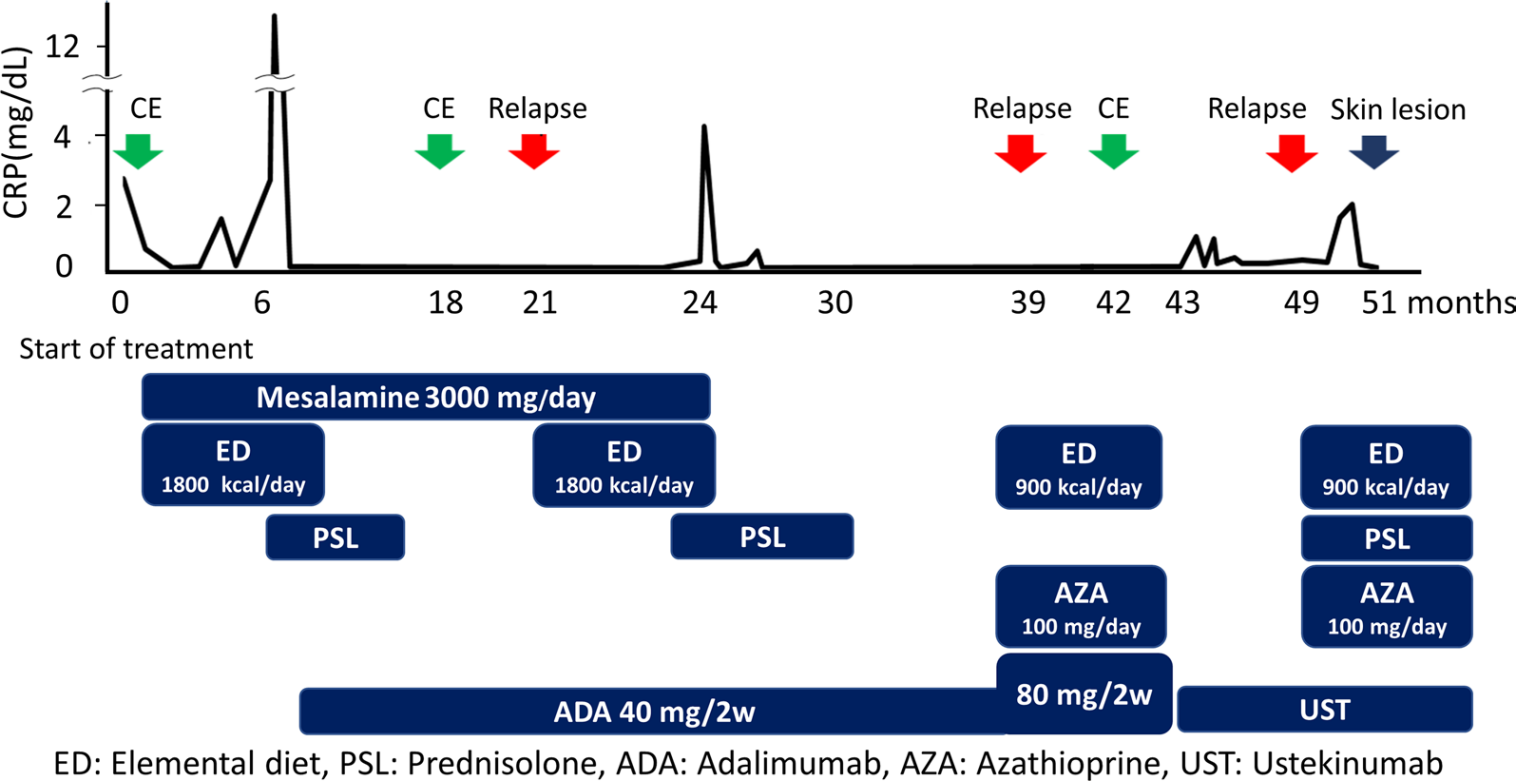
Entire clinical course of the present case

**Fig. 3:**
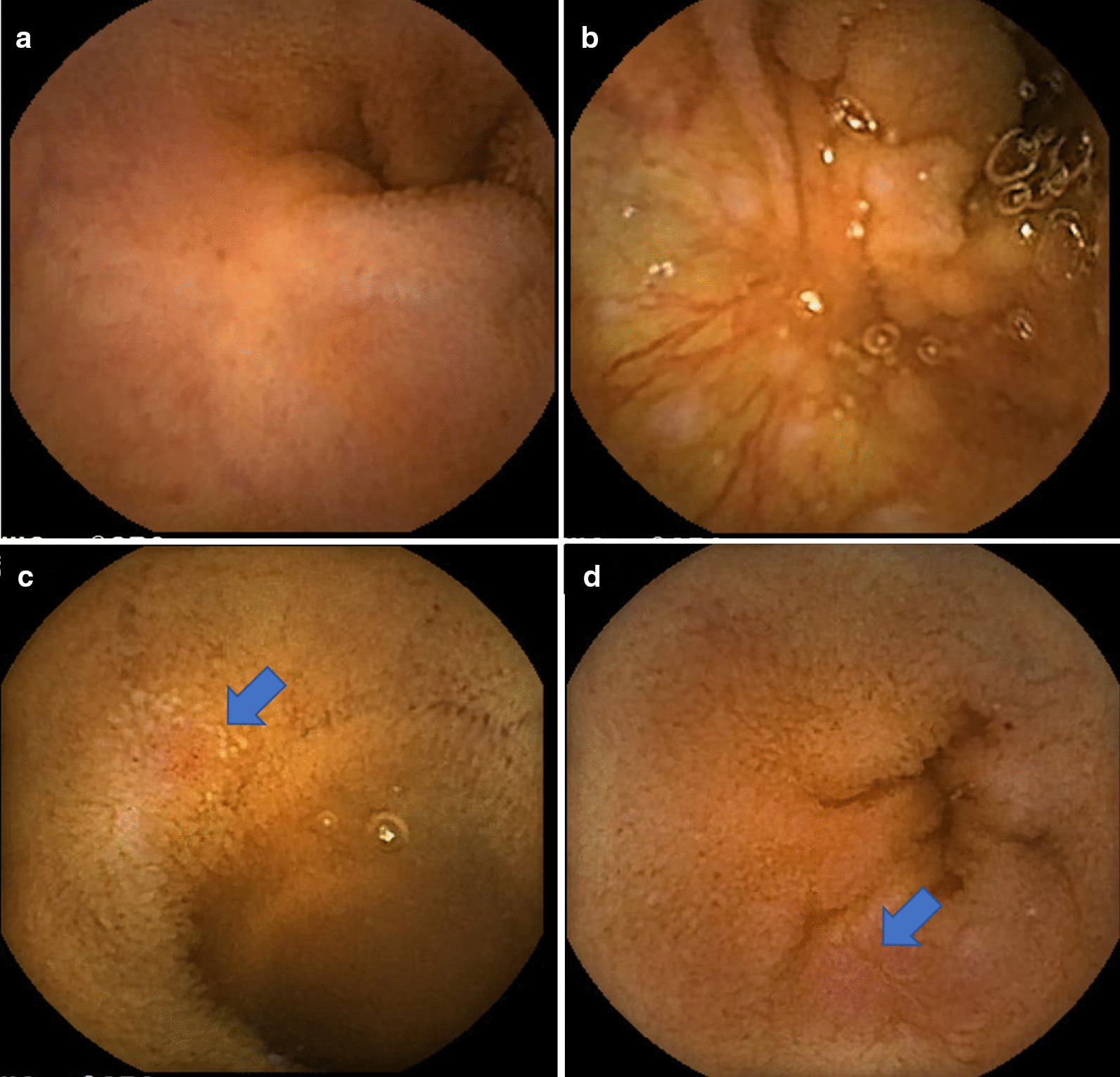
Findings of the second capsule endoscopic examination. **a**, **b** Ulcer scars on the jejunum and ileum (18 months after the first treatment). **c**, **d** Small recurrent erosions on the jejunum and ileum (42 months after the first treatment)

**Fig. 4:**
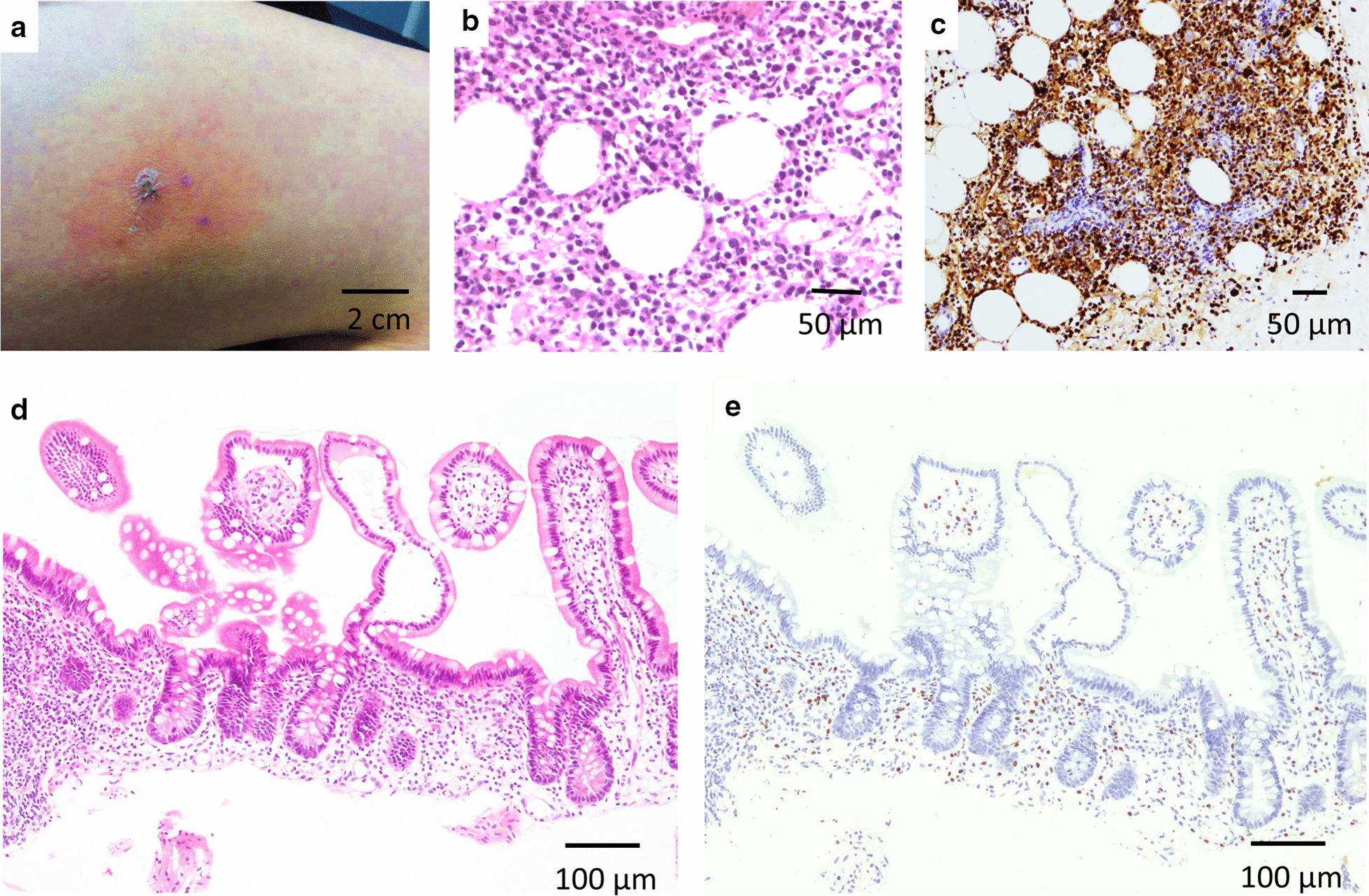
Histological findings of extranodal NK/T-cell lymphoma (nasal type) associated with continuously activated EBV infection. **a** Macroscopic finding of skin ulcer on the patient’s thigh, which occurred more than 4 years after the initiation of medical treatment. **b** Microscopic finding of H&E staining of the skin ulcer. **c** Microscopic findings revealed high proliferation of EBER-positive atypical lymphocytes in the diseased skin. **d** Microscopic finding of H&E staining in the intestinal ulcer. **e** Microscopic immunohistological findings of ileal ulcerative lesions revealed moderate infiltration of many EBER-positive lymphocytes, which demonstrated continuously active EBV infection

**Fig. 5:**
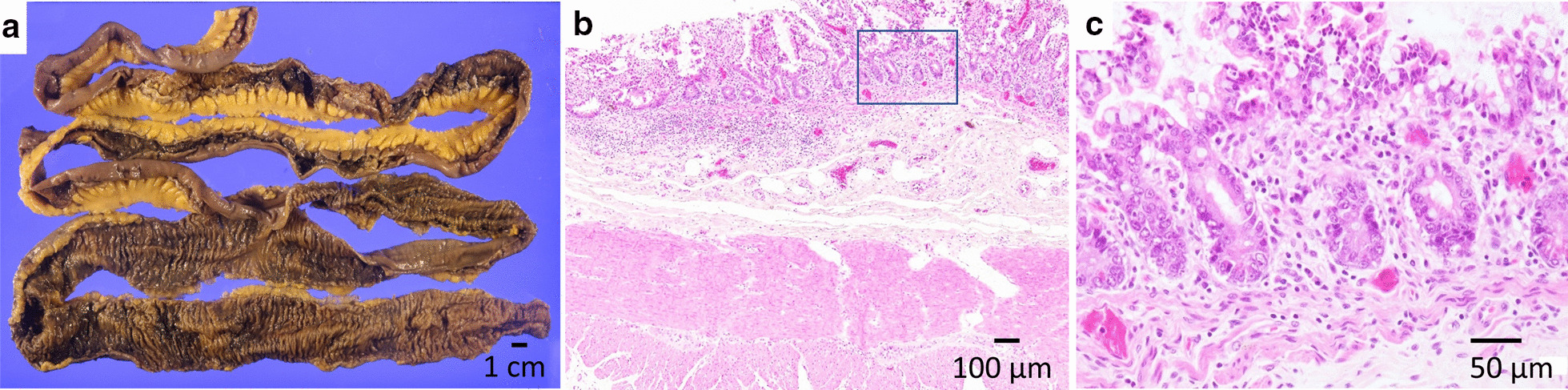
Histological findings of the autopsied small intestine. **a** Macroscopic observation revealed neither ulcerative changes nor stenotic changes in the small intestine. **b** Microscopic finding of H&E staining of the intestinal tissue. **c** Microscopic observation revealed mild lymphocyte proliferation with no atrophy of the intestinal villi (large magnification of H&E staining)

A 19-year-old woman was referred to our hospital for intermittent abdominal pain and continuous fever which had persisted for a month. She had no obvious medical or familial history. Physical examination revealed no morbid lymph node swelling or skin abnormalities. Blood examination showed normal blood cell counts and no presence of atypical lymphocytes. Biochemical or immunoserum analysis revealed modest inflammation, hypoalbuminemia, slight liver dysfunction, and evidence of past EBV infection (Additional file 1: Table S1). Although esophagogastroduodenoscopy revealed normal findings, colonoscopy revealed a few small ulcers in the terminal ileum (Additional file 3: Fig. 1). Enhanced computed tomography showed obvious wall thickness of the small intestine, whereas no significant finding was detected by balloon intestinal endoscopy in the distal ileum. However, various types of ulcers surrounded by completely normal mucosa were found on the jejunum and proximal ileum by capsule endoscopy (CE) (Fig. 1). Additionally, multiple aphthous and some linear ulcers were observed on the jejunum, whereas circular ulcers and longitudinal ulcers with a cobblestone appearance were detected on the ileum. The possibility of intestinal tuberculosis and infectious gastroenteritis were serologically and culturally excluded. Although there were no histological findings of noncaseating granuloma on her digestive organs, she was strongly suspected with CD based on her age, clinical symptoms, and the morphology of the characteristic ulcers (Fig. 2). Consequently, the patient was treated with an elemental diet, mesalamine, and prednisolone for induction therapy. Although this treatment seemed to be partially effective, her symptoms recurred according to the tapering of prednisolone, and she was subsequently switched to adalimumab (ADA). Her clinical symptoms tended to be gradually modest, and the maintenance ADA therapy was effective for some time. The second CE revealed a definite improvement of the intestinal ulcers (Fig. 3a, b); however, 21 months after the initiation of treatment, her intermittent fever and repeated abdominal pain recurred, and re-induction therapy with prednisolone was restarted (Fig. 2). She remained stable for 9 months, before complaining of slight abdominal pain. We set a dose escalation of ADA with the addition of azathioprine; this seemed to be effective, and almost all of the ulcers disappeared, except for a few small erosions on the intestine (Fig. 3c, d). She next relapsed approximately 10 months later, and ADA was switched to ustekinumab (USK). However, this treatment was not sufficient, and prednisolone was added to USK for clinical improvement. Unfortunately, disease management was difficult regardless of these treatments, and skin ulcers appeared about 50 months after her initial hospital visit (Fig. 4a). Immunohistology of her skin biopsy revealed proliferation of EBV-encoded small RNA (EBER)-positive atypical lymphocytes (Fig. 4b, c). Retrospective assessments of the previous ileal ulcer biopsy before treatment demonstrated many EBER-positive lymphocytes, indicating that she had been in a continuously active EBV infection state (Fig. 4d, e). The blood level of EBV-DNA was also clearly positive (1.5 × 10^4^ copy/WBC × 10^6^). Consequently, she was diagnosed with extranodal NK/T-cell lymphoma (nasal type) related to CAEBV. Advanced-stage malignant lymphoma invading the skin was seen. Then, she was treated with a combination of cyclosporine A and prednisolone for her systemic inflammation related to CAEBV and followed by a systemic chemotherapy for malignant lymphoma. After completing the CHOP chemotherapy (cyclophosphamide, doxorubicin, vincristine, and prednisolone), she gradually showed intermediate liver injury as well as cholestasis, based on which she was diagnosed with secondary hemophagocytic lymphohistiocytosis (HLH) caused by CAEBV. Though etoposide and methylprednisolone combination therapy transiently improved her liver injury and jaundice, CHOP insufficiently controlled the progression of CAEBV. Hence, the combination therapy of etoposide, cytosine arabinoside, l-asparaginase, methylprednisolone, and prednisolone (ESCAP) was preferred over the CHOP therapy. However, her liver injury and jaundice related to HLH rapidly worsened during the recovery period after the first ESCAP therapy. Unfortunately, her disease progression could not be regulated at all and she finally fell into the stage of multiple organ failure; she died at the age of 23 years, 55 months after her first hospital visit. An autopsy was performed with the consent from her parents. Macroscopic findings showed no evidence of ulcer or stenotic changes on her digestive organs, including the small intestine (Fig. 5a). Microscopic findings showed no evidence of noncaseating granuloma on digestive organs and a remarkable proliferation of lymphocytes in the intra-mucosal/submucosal space of the small intestine (Fig. 5b, c).

## Discussion and conclusion

Chachu and Osterman concluded that "Thoughtful consideration and evaluation of these other potential etiologies through patient history and physical examination, laboratory tests, endoscopic evaluation with targeted biopsies of lesions, and cross-sectional imaging is required to evaluate any patient presenting with symptoms consistent with IBD" [7]. However, it is difficult to completely distinguish CAEBV enteritis from IBD by endoscopic findings only, and it is important to comprehend the clinical features of each disease. To appropriately diagnose CAEBV, it is important to remember the possibility of CAEBV as a differential diagnosis of systemic inflammation [8]. When CAEBV is suspected, the EBV-DNA level in peripheral blood should be checked, even in immunologically denied cases of acute EBV infection. Identification of EBV-infected T and NK cells should be performed for definite diagnosis of CAEBV. Moreover, in situ hybridization (ISH) of EBER by pathological examination and EBV-DNA analysis by flow cytometry should be performed. More details are described in the latest diagnostic criteria of CAEBV [5, 9].

To our knowledge, the first report of a case with gastrointestinal tract complications of CAEBV had serious colorectal bleeding [10]. Later, in the 2000s, there were several reports of CAEBV with gastrointestinal tract complications, which had initially been misdiagnosed as IBD [2–4]. Approximately 6% CAEBV-related deaths originated from intestinal bleeding or perforation [6]. As summarized in the Additional file 2: Table S2, the ulcer morphology of “cobblestone appearance” characteristic of CD [11] was first found in our current case, but this was not observed in all 27 previously reported CAEBV-related enteritis cases [2–4, 10, 12, 13]. According to the endoscopic features of these 28 cases, the diseased lesion was located in the colon (13 cases), small intestine (five cases), concomitant colon and small intestine (five cases), concomitant colon and ileocecal junction (one case), concomitant colon and stomach (one case), ileocecal junction (one case), and unknown location (two cases). Ulcer morphologies have generally been reported as small, shallow, irregular-shaped, and scattered, with the exception of eight cases of huge and profound ulcers. There was also a previous case in which the entire intestinal mucosa displayed lymphangiectasia [3]. In our case, many small aphthous ulcers were found in the whole intestine, and a cobblestone appearance was observed, especially in the ileum, whereas no ulcer was found in the colon. These aphthous ulcers remained after intensive treatment with steroids and biologics. Based on these findings, CAEBV-related enteritis might present various ulcer morphologies depending on the reaction of each therapeutic treatment and/or time course. To detect intestinal lesions, we adopted CE, which has been increasingly used worldwide since its establishment in 2000 [14]. With the advent of CE, various diseases have been clearly visualized, resulting in a paradigm shift in the diagnosis and treatment of small bowel disease. According to a nationwide study in Japan, the frequent findings of CE in patients with CD include cobblestone appearance (occurrence rate: 33%), longitudinal ulcer (78%), irregular ulcer (84%), liner erosion (90%), irregular erosion (89%), circumferential alignment of diminutive lesions (75%), and longitudinal alignment of diminutive lesions (56%) [15]. In this previous study, Esaki et al. observed that the endoscopic diagnosis varied in endoscopist’s clinical knowledge and proficiency; hence, this issue should be overcome. In this case, CD was most probable because ulcers with a cobblestone appearance were detected in the ileum. However, other characteristic findings, such as the bamboo joint-like appearance of the gastric mucosa in esophagogastroduodenoscopy, focally enhanced gastritis, and granuloma on histological evaluation were not actually confirmed.

Thus, a final diagnosis should be comprehensively made based on physical symptoms, medical histories, clinical findings, and culture, imaging, and pathological tests. Even if the characteristic endoscopic findings of specific enteritis are detected, differential diagnosis based only on these findings will be difficult because the characteristic findings of endoscopy do not always correspond to the specific disease. Some enteric diseases might show similar endoscopic appearances, whereas the same disease might demonstrate various phenotypes depending on the time course and severity.

Unfortunately, no successful treatment for CAEBV has yet been established, and further research is needed to improve the outcome in the future [16, 17]. Therefore, it is crucial for clinicians to definitely diagnose CAEBV as quickly as possible.

In conclusion, we diagnosed a rare case of CAEBV, with refractory enteritis as the main clinical symptom and without manifestation of a typical IM feature. Diagnosing CAEBV by ulcer morphology is difficult, even with CE and routine histopathological examination only; thus, clinicians should consider CAEBV as a differential diagnosis of refractory enteritis in younger patients and eagerly check blood EBV-DNA and EBER expression levels by ISH.

## Patient perspective

The patient's mother kindly told the authors that the patient had decided to fight her disease, though she had well realized the features and general prognosis of CAEBV.

## Supplementary Information

**Additional file 1: Supplementary Table S1**. Results of her blood test on the first admission.

**Additional file 2: Supplementary Table S2.** Clinical features of the reported CAEBV cases.

**Additional file 3**: **Figure S1.** Endoscopic findings of each digestive organ before treatment. (a) Esophagus, (b) stomach, (c) duodenum, (d) terminal ileum, (e) colon, and (f) rectum. Arrow indicates an ulcerative lesion on the terminal ileum.

## Data Availability

Data on this case not reported in the manuscript are available from the corresponding author upon reasonable request.

## Abbreviations

CAEBV: Chronic active Epstein–Barr virus infection
EBV: Epstein–Barr virus
CD: Crohn’s disease
IBD: Inflammatory bowel disease
IM: Infectious mononucleosis
CE: Capsule endoscopy
ADA: Adalimumab
USK: Ustekinumab
EBER: EBV-encoded small mRNA
ISH: In situ hybridization
HLH: Hemophagocytic lymphohistiocytosis
