# Supplementary material for: Chronic active EBV infection in refractory enteritis with longitudinal ulcers with a cobblestone appearance: an autopsied case report
Source: BMC Gastroenterol. 2021 Jan 6;21:6. doi: 10.1186/s12876-020-01589-1 (PMC7789587; doi:10.1186/s12876-020-01589-1)
Supplement: Supplementary file 1 — Additional file 1: Supplementary Table S1. Results of her blood test on the first admission. [file 12876_2020_1589_MOESM1_ESM.docx]

Supplementary Table 1

| **White blood cell** | **6000　/μL** | **ESR 1h** | **21　mm** | **Total cholesterol** | **177　mg/dL** | **IgM-Hepatitis A Ab** | **0.18　S/CO** |
| --- | --- | --- | --- | --- | --- | --- | --- |
| **Neutrophil** | **73.8%** | **ESR 2h** | **52　mm** | **Triglyceride** | **383　mg/dL** | **Hepatitis B surface Ag** | **0.01　U/mL** |
| **Lymphocyte** | **15.7%** | **PT** | **111%** | **Uric acid** | **3.59　mg/dL** | **IgM-Hepatitis B core Ab** | **0.1　S/CO** |
| **Monocyte** | **5.1%** | **PT-INR** | **1.07　mg/dL** | **Glucose** | **88　mg/dL** | **IgG-Hepatitis B core Ab** | **0.01　U/mL** |
| **Eosinophil** | **1.6%** | **Total bilirubin** | **0.47　mg/dL** | **Total protein** | **5.8g/dL** | **Hepatitis C virus Ab** | **0.1　S/CO** |
| **Basophil** | **0.4 %** | **Direct bilirubin** | **0.04　U/L** | **Albumin** | **3.2　g/dL** | **Cytomegalovirus IgM** | **0.44　S/CO** |
| **Atypical lymphocyte** | **0%** | **Aspartate transaminase** | **41　U/L** | **C-reactive protein** | **2.75　mg/dL** | **Cytomegalovirus IgG** | **51.9　U/mL** |
| **Red blood cell** | **468×10^4^　/μL** | **Alanine aminotransferase** | **33　U/L** | **Blood　urea　nitrogen** | **5.9　mg/dL** | **Epstein-Barr virus capsid antigen IgM** | **＜× 10** |
| **Hemoglobin** | **13.8　g/dL** | **Lactate dehydrogenase** | **284　U/L** | **Creatinine** | **0.51　mg/dL** | **Epstein-Barr viruscapsid antigen IgG** | **× 40　U/mL** |
| **Hematocrit** | **41.3%** | **Alkaline phosphatase** | **207　U/L** | **Sodium** | **141　mEq/L** | **Epstein-Barr virus nuclear antigen** | **× 40　S/CO** |
| **MCV** | **88.2　fL** | **γ-glutamyl transpeptidase** | **20　U/L** | **Potassium** | **4.5　mEq/L** |  |  |
| **MCH** | **29.5　pg** | **Cholinesterase** | **255　U/L** | **Chloride** | **105　mEq/L** |  |  |
| **MCHC** | **33.4%** | **Creatinine kinase** | **44　U/L** | **Calcium** | **8.9　mEq/L** |  |  |
| **Platelet** | **20.8×10^4^/μL** | **Amylase** | **88　mm** | **Ferrous** | **78　μg/dL** |  |  |
|  |  |  |  | **Ferritin** | **272　ng/mL** |  |  |
|  |  |  |  | **Total cholesterol** | **177　mg/dL** |  |  |

MCV: Mean corpuscular volume; MCH: Mean corpuscular hemoglobin; MCHC: Mean corpuscular hemoglobin concentration; ESR: Erythrocyte sedimentation rate; PT: Prothrombin time; PT-INR: Prothrombin time international normalized ratio; Ag: Antigen; Ab: Antibody; Ig: Immunoglobulin.
