# Supplementary material for: Chronic active EBV infection in refractory enteritis with longitudinal ulcers with a cobblestone appearance: an autopsied case report
Source: BMC Gastroenterol. 2021 Jan 6;21:6. doi: 10.1186/s12876-020-01589-1 (PMC7789587; doi:10.1186/s12876-020-01589-1)
Supplement: Supplementary file 2 — Additional file 2: Supplementary Table S2. Clinical features of the reported CAEBV cases. [file 12876_2020_1589_MOESM2_ESM.docx]

| Sex | Male: 22 cases, Female: 5 cases, unknown: 1 case. |
| --- | --- |
| Median age (range) | 9<: 1 case, 10s: 3 cases 20s: 4cases, 30s: 4 cases 40s: 6 cases, 50s: 6 cases, 60s: none, 70s: 3 cases, unknown: 1 case |
| Location of enteritis | colon (13 cases), small intestine (5 cases), concomitant of colon and small intestine (5 cases), concomitant of colon and ileocecal junction (1 case), concomitant of colon and stomach (1 case), ileocecal junction (1 case), and details unknown (2 cases). |
| Morphology of ulcer | shallow or small: 14 cases, huge or profound: 8 cases, irregular: 6 cases, unknown: 1 case. |
| Longitudinal ulcers with a cobble stone appearance | Our case (only one case). |
| Special type | Lymphangiectasis in whole small intestine (only one case). |
