# Supplementary figures and images for: Chronic active EBV infection in refractory enteritis with longitudinal ulcers with a cobblestone appearance: an autopsied case report
Source: BMC Gastroenterol. 2021 Jan 6;21:6. doi: 10.1186/s12876-020-01589-1 (PMC7789587; doi:10.1186/s12876-020-01589-1)

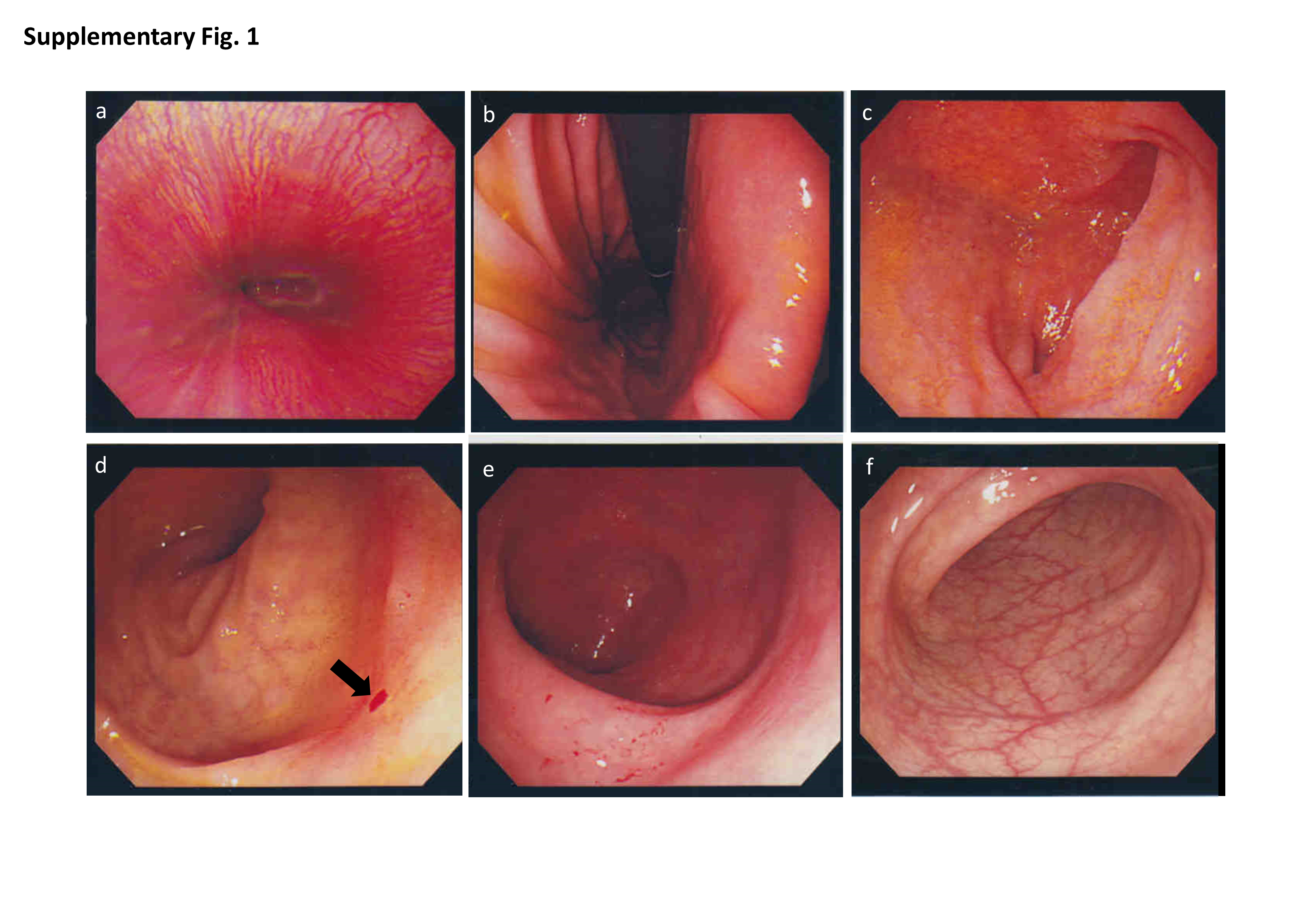

Supplement: Supplementary file 3 — Additional file 3: Figure S1. Endoscopic findings of each digestive organ before treatment. (a) Esophagus, (b) stomach, (c) duodenum, (d) terminal ileum, (e) colon, and (f) rectum. Arrow indicates an ulcerative lesion on the terminal ileum. [file 12876_2020_1589_MOESM3_ESM.tif]
